# *catena*-Poly[[bis­[2-chloro-6-(1*H*-1,2,4-triazol-1-yl-κ*N* ^4^)pyridine]cadmium(II)]-di-μ-thio­cyanato-κ^2^ *N*:*S*;κ^2^ *S*:*N*]: a one-dimensional coordination polymer

**DOI:** 10.1107/S1600536808030547

**Published:** 2008-09-27

**Authors:** Zhong Nian Yang, Ting Ting Sun

**Affiliations:** a Department of Chemistry and Chemical Engineering, Binzhou University, Binzhou 256603, People’s Republic of China; b Department of Chemistry, Shandong Normal University, Jinan 250014, People’s Republic of China

## Abstract

In the crystal structure of the title complex, [Cd(NCS)_2_(C_7_H_5_ClN_4_)_2_]_*n*_, the Cd^II^ atom lies on a crystallographic inversion center and assumes a distorted octa­hedral geometry. The 2-chloro-6-(1*H*-1,2,4-triazol-1-yl)pyridine mol­ecule acts as a terminal ligand. The thio­cyanate ligands function as μ_1,3_-bridging units connecting adjacent Cd^II^ atoms with a separation of 5.7525 (11) Å, forming a one-dimensional chain along the *a* axis.

## Related literature

For a related structure, see: Shi *et al.* (2006 ▶).
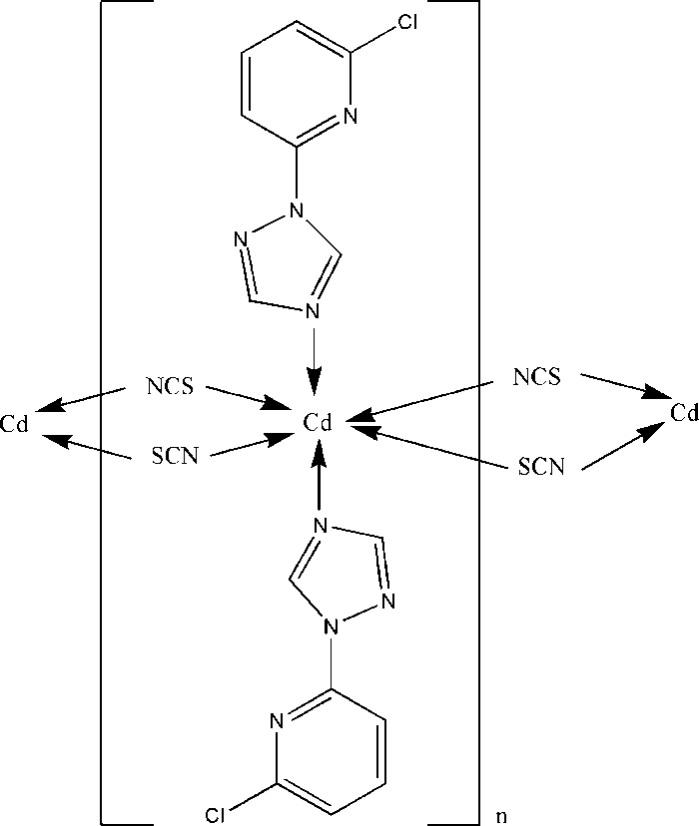


## Experimental

### 

#### Crystal data

[Cd(NCS)_2_(C_7_H_5_ClN_4_)_2_]
*M* *_r_* = 589.76
Triclinic, 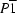

*a* = 5.7525 (11) Å
*b* = 8.0180 (15) Å
*c* = 12.212 (2) Å
α = 107.609 (3)°
β = 90.095 (2)°
γ = 91.950 (3)°
*V* = 536.53 (18) Å^3^
*Z* = 1
Mo *K*α radiation
μ = 1.49 mm^−1^
*T* = 298 (2) K
0.23 × 0.21 × 0.10 mm

#### Data collection

Bruker SMART APEX CCD diffractometer
Absorption correction: multi-scan (**SADABS**; Sheldrick, 1996 ▶) *T*_min_ = 0.726, *T*_max_ = 0.865
2892 measured reflections
2005 independent reflections
1903 reflections with *I* > 2σ(*I*)
*R*_int_ = 0.016

#### Refinement

*R*[*F* ^2^ > 2σ(*F* ^2^)] = 0.028
*wR*(*F* ^2^) = 0.071
*S* = 1.03
2005 reflections
143 parameters
H-atom parameters constrained
Δρ_max_ = 0.34 e Å^−3^
Δρ_min_ = −0.36 e Å^−3^

### 

Data collection: *SMART* (Bruker, 1997 ▶); cell refinement: *SAINT* (Bruker, 1997 ▶); data reduction: *SAINT*; program(s) used to solve structure: *SHELXS97* (Sheldrick, 2008 ▶); program(s) used to refine structure: *SHELXL97* (Sheldrick, 2008 ▶); molecular graphics: *SHELXTL* (Sheldrick, 2008 ▶); software used to prepare material for publication: *SHELXTL*.

## Supplementary Material

Crystal structure: contains datablocks I, global. DOI: 10.1107/S1600536808030547/is2339sup1.cif

Structure factors: contains datablocks I. DOI: 10.1107/S1600536808030547/is2339Isup2.hkl

Additional supplementary materials:  crystallographic information; 3D view; checkCIF report

## Figures and Tables

**Table:** 

Cd1—N5	2.319 (2)
Cd1—N1	2.328 (2)
Cd1—S1^i^	2.7696 (9)

**Table:** 

N5^ii^—Cd1—N1	89.51 (9)
N5—Cd1—N1	90.49 (9)
N5—Cd1—S1^i^	88.71 (7)
N1—Cd1—S1^i^	90.02 (6)
N5—Cd1—S1^iii^	91.29 (7)
N1—Cd1—S1^iii^	89.98 (6)

Symmetry codes: (i) 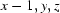; (ii) 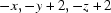; (iii) 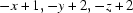.

**Fig. 1.**
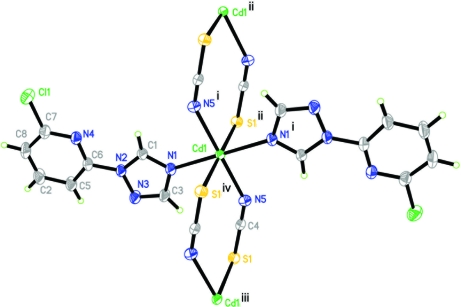
View of complex (I), showing the atom numbering scheme with displacement ellipsoids drawn at the 30% probability level [symmetry codes: (i) -x, -y + 2, -z + 2; (ii) x - 1, y, -z + 1; (iii) x + 1, y, z; (iv) -x + 1, -y + 2, -z + 2].

**Fig. 2.**
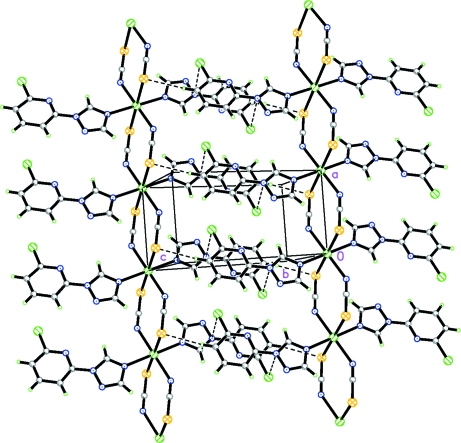
Packing diagram of (I), showing one-dimensional chains.

## supplementary crystallographic information

### Comment

For a long time, thiocyanate anion has been used as a bridge ligand and a number of complexes with it have been published (Shi *et al.*, 2006). But complex dealing with 2-chloro-6-(1*H*-1,2,4-triazol-1-yl)pyridine as a ligand has not been reported as yet as our knowledge. The interest in complexes with mixed bridge ligands resulted in us synthesizing the title complex and here we report its crystal structure, (I).

The asymmetric unit and symmetry-related fragments of (I) are shown in Fig. 1. Atom Cd1 is located on an inversion center and is in a distorted octahedral CdN_4_S_2_ coordination geometry (Table 1). In the crystal 2-chloro-6-(1*H*-1,2,4-triazol-1-yl)pyridine molecule only acts as a unidentate terminal ligand, and thiocyanate anion functions as a µ-1,3 bridge ligand and joins a pair of Cd^II^ ions with separation of 5.7525 (11) Å. In this way a one-dimensional chain along *a* axis was fabricated as shown in Fig. 2. In addition, there is a weak π-π stacking interaction involving symmetry related 2-chloro-6-(1*H*-1,2,4-triazol-1-yl)pyridine molecules, with relevant distances being *Cg*1···*Cg*2^i^ = 3.7095 (19) Å, *Cg*1···*Cg*2^i^_perp_ = 3.427 Å, α = 4.24° [symmetry code: (i) -*x*, 2-*y*,1-*z*; *Cg*1 and *Cg*2 are the centroids of the pyrazole ring and pyridyl ring, respectively; *Cg*1···*Cg*2^i^_perp_ is the perpendicular distance from ring *Cg*1 to ring *Cg*2^i^; α is the dihedral angle between plane *Cg*1 and plane *Cg*2^i^.

### Experimental

15 ml H_2_O solution containing Cd(ClO_4_)_2_.6H_2_O (0.1507 g, 0.359 mmol) and NaSCN (0.0591 g, 0.729 mmol) was added into 15 ml methanol solution of 2-chloro-6-(1*H*-1,2,4-triazol-1-yl)pyridine (0.1203 g, 0.666 mmol), and the mixed solution was stirred for a few minutes. The colorless single crystals were obtained after the filtrate had been allowed to stand at room temperature for about three weeks.

### Refinement

All H atoms were placed in calculated positions (C—H = 0.93 Å) and refined as riding, with *U*_iso_(H) = 1.2 *U*_eq_(C).

### Crystal data

**Table:**

[Cd(NCS)_2_(C_7_H_5_ClN_4_)_2_]	*Z* = 1
*M**_r_* = 589.76	*F*(000) = 290
Triclinic, *P*1	*D*_x_ = 1.825 Mg m^−^^3^
Hall symbol: -P 1	Mo *K*α radiation, λ = 0.71073 Å
*a* = 5.7525 (11) Å	Cell parameters from 1921 reflections
*b* = 8.0180 (15) Å	θ = 2.7–28.1°
*c* = 12.212 (2) Å	µ = 1.49 mm^−^^1^
α = 107.609 (3)°	*T* = 298 K
β = 90.095 (2)°	Block, colorless
γ = 91.950 (3)°	0.23 × 0.21 × 0.10 mm
*V* = 536.53 (18) Å^3^	

### Data collection

**Table:**

Bruker SMART APEX CCD diffractometer	2005 independent reflections
Radiation source: fine-focus sealed tube	1903 reflections with *I* > 2σ(*I*)
graphite	*R*_int_ = 0.016
φ and ω scans	θ_max_ = 25.8°, θ_min_ = 1.8°
Absorption correction: multi-scan (*SADABS*; Sheldrick, 1996)	*h* = −6→7
*T*_min_ = 0.726, *T*_max_ = 0.865	*k* = −9→8
2892 measured reflections	*l* = −11→14

### Refinement

**Table:**

Refinement on *F*^2^	Secondary atom site location: difference Fourier map
Least-squares matrix: full	Hydrogen site location: inferred from neighbouring sites
*R*[*F*^2^ > 2σ(*F*^2^)] = 0.028	H-atom parameters constrained
*wR*(*F*^2^) = 0.071	*w* = 1/[σ^2^(*F*_o_^2^) + (0.0366*P*)^2^ + 0.1572*P*] where *P* = (*F*_o_^2^ + 2*F*_c_^2^)/3
*S* = 1.03	(Δ/σ)_max_ = 0.002
2005 reflections	Δρ_max_ = 0.34 e Å^−^^3^
143 parameters	Δρ_min_ = −0.36 e Å^−^^3^
0 restraints	Extinction correction: SHELXL97 (Sheldrick, 2008), Fc^*^=kFc[1+0.001xFc^2^λ^3^/sin(2θ)]^-1/4^
Primary atom site location: structure-invariant direct methods	Extinction coefficient: 0.021 (2)

### Special details

**Table:**

Geometry. All e.s.d.'s (except the e.s.d. in the dihedral angle between two l.s. planes) are estimated using the full covariance matrix. The cell e.s.d.'s are taken into account individually in the estimation of e.s.d.'s in distances, angles and torsion angles; correlations between e.s.d.'s in cell parameters are only used when they are defined by crystal symmetry. An approximate (isotropic) treatment of cell e.s.d.'s is used for estimating e.s.d.'s involving l.s. planes.
Refinement. Refinement of *F*^2^ against ALL reflections. The weighted *R*-factor *wR* and goodness of fit *S* are based on *F*^2^, conventional *R*-factors *R* are based on *F*, with *F* set to zero for negative *F*^2^. The threshold expression of *F*^2^ > σ(*F*^2^) is used only for calculating *R*-factors(gt) *etc*. and is not relevant to the choice of reflections for refinement. *R*-factors based on *F*^2^ are statistically about twice as large as those based on *F*, and *R*- factors based on ALL data will be even larger.

### Fractional atomic coordinates and isotropic or equivalent isotropic displacement parameters (Å^2^)

**Table:**

	*x*	*y*	*z*	*U*_iso_*/*U*_eq_	
C1	−0.0042 (5)	0.8697 (4)	0.7162 (2)	0.0411 (6)	
H1	−0.1436	0.8065	0.7139	0.049*	
C2	0.1609 (7)	0.7193 (5)	0.3111 (3)	0.0639 (9)	
H2	0.2549	0.7310	0.2518	0.077*	
C3	0.2948 (5)	1.0298 (4)	0.7697 (2)	0.0534 (8)	
H3	0.4058	1.1035	0.8178	0.064*	
C4	0.5043 (5)	1.2276 (4)	1.0409 (2)	0.0421 (6)	
C5	0.2157 (6)	0.8143 (4)	0.4239 (2)	0.0543 (8)	
H5	0.3444	0.8913	0.4426	0.065*	
C6	0.0695 (5)	0.7883 (3)	0.5063 (2)	0.0406 (6)	
C7	−0.1629 (6)	0.5969 (4)	0.3779 (2)	0.0484 (7)	
C8	−0.0303 (7)	0.6092 (4)	0.2872 (2)	0.0597 (8)	
H8	−0.0696	0.5446	0.2121	0.072*	
Cd1	0.0000	1.0000	1.0000	0.04300 (14)	
Cl1	−0.40595 (17)	0.45711 (12)	0.35184 (8)	0.0709 (3)	
N1	0.1037 (4)	0.9656 (3)	0.81047 (18)	0.0446 (5)	
N2	0.1161 (4)	0.8768 (3)	0.62460 (17)	0.0401 (5)	
N3	0.3112 (4)	0.9806 (3)	0.65819 (19)	0.0530 (6)	
N4	−0.1181 (4)	0.6839 (3)	0.48723 (18)	0.0420 (5)	
N5	0.3274 (4)	1.1798 (4)	1.0652 (2)	0.0538 (6)	
S1	0.75769 (13)	1.29435 (10)	1.00393 (6)	0.0488 (2)	

### Atomic displacement parameters (Å^2^)

**Table:**

	*U*^11^	*U*^22^	*U*^33^	*U*^12^	*U*^13^	*U*^23^
C1	0.0387 (14)	0.0535 (15)	0.0288 (12)	−0.0045 (11)	0.0033 (10)	0.0098 (11)
C2	0.081 (2)	0.078 (2)	0.0301 (15)	−0.0033 (18)	0.0091 (14)	0.0133 (14)
C3	0.0507 (17)	0.073 (2)	0.0300 (13)	−0.0198 (15)	0.0021 (12)	0.0083 (13)
C4	0.0382 (15)	0.0524 (16)	0.0289 (12)	−0.0036 (12)	−0.0049 (10)	0.0026 (11)
C5	0.065 (2)	0.0628 (19)	0.0318 (14)	−0.0065 (15)	0.0078 (13)	0.0112 (13)
C6	0.0517 (16)	0.0411 (14)	0.0278 (12)	0.0034 (12)	0.0011 (11)	0.0084 (10)
C7	0.0575 (18)	0.0419 (15)	0.0413 (15)	0.0064 (12)	−0.0075 (13)	0.0052 (11)
C8	0.083 (2)	0.0602 (19)	0.0292 (14)	0.0067 (17)	−0.0040 (14)	0.0025 (13)
Cd1	0.02956 (18)	0.0703 (2)	0.02358 (16)	−0.01005 (12)	0.00224 (10)	0.00706 (12)
Cl1	0.0652 (6)	0.0672 (5)	0.0659 (5)	−0.0086 (4)	−0.0153 (4)	0.0000 (4)
N1	0.0437 (13)	0.0585 (14)	0.0279 (11)	−0.0047 (10)	0.0042 (9)	0.0085 (10)
N2	0.0434 (13)	0.0482 (12)	0.0263 (10)	−0.0039 (10)	0.0034 (9)	0.0082 (9)
N3	0.0533 (15)	0.0698 (16)	0.0312 (12)	−0.0195 (12)	0.0052 (10)	0.0105 (11)
N4	0.0487 (14)	0.0434 (12)	0.0321 (11)	0.0048 (10)	−0.0018 (9)	0.0084 (9)
N5	0.0360 (14)	0.0676 (16)	0.0479 (14)	−0.0086 (11)	0.0004 (10)	0.0035 (12)
S1	0.0407 (4)	0.0620 (5)	0.0401 (4)	−0.0104 (3)	0.0037 (3)	0.0113 (3)

### Geometric parameters (Å, °)

**Table:**

C1—N1	1.316 (3)	C6—N2	1.425 (3)
C1—N2	1.331 (3)	C7—N4	1.326 (3)
C1—H1	0.9300	C7—C8	1.372 (5)
C2—C8	1.361 (5)	C7—Cl1	1.729 (3)
C2—C5	1.387 (4)	C8—H8	0.9300
C2—H2	0.9300	Cd1—N5^i^	2.319 (2)
C3—N3	1.303 (3)	Cd1—N5	2.319 (2)
C3—N1	1.356 (3)	Cd1—N1	2.328 (2)
C3—H3	0.9300	Cd1—N1^i^	2.328 (2)
C4—N5	1.146 (4)	Cd1—S1^ii^	2.7696 (9)
C4—S1	1.646 (3)	Cd1—S1^iii^	2.7696 (9)
C5—C6	1.371 (4)	N2—N3	1.360 (3)
C5—H5	0.9300	S1—Cd1^iv^	2.7696 (9)
C6—N4	1.319 (4)		
			
N1—C1—N2	109.8 (2)	N5—Cd1—N1	90.49 (9)
N1—C1—H1	125.1	N5^i^—Cd1—N1^i^	90.49 (9)
N2—C1—H1	125.1	N5—Cd1—N1^i^	89.51 (9)
C8—C2—C5	120.1 (3)	N1—Cd1—N1^i^	180.000 (1)
C8—C2—H2	120.0	N5^i^—Cd1—S1^ii^	91.29 (7)
C5—C2—H2	120.0	N5—Cd1—S1^ii^	88.71 (7)
N3—C3—N1	115.0 (3)	N1—Cd1—S1^ii^	90.02 (6)
N3—C3—H3	122.5	N1^i^—Cd1—S1^ii^	89.98 (6)
N1—C3—H3	122.5	N5^i^—Cd1—S1^iii^	88.71 (7)
N5—C4—S1	179.1 (3)	N5—Cd1—S1^iii^	91.29 (7)
C6—C5—C2	116.3 (3)	N1—Cd1—S1^iii^	89.98 (6)
C6—C5—H5	121.9	N1^i^—Cd1—S1^iii^	90.02 (6)
C2—C5—H5	121.9	S1^ii^—Cd1—S1^iii^	180.0
N4—C6—C5	125.8 (3)	C1—N1—C3	103.0 (2)
N4—C6—N2	114.1 (2)	C1—N1—Cd1	127.88 (18)
C5—C6—N2	120.1 (3)	C3—N1—Cd1	129.05 (18)
N4—C7—C8	124.9 (3)	C1—N2—N3	110.0 (2)
N4—C7—Cl1	115.9 (2)	C1—N2—C6	129.0 (2)
C8—C7—Cl1	119.2 (2)	N3—N2—C6	120.9 (2)
C2—C8—C7	117.6 (3)	C3—N3—N2	102.2 (2)
C2—C8—H8	121.2	C6—N4—C7	115.4 (2)
C7—C8—H8	121.2	C4—N5—Cd1	145.7 (2)
N5^i^—Cd1—N5	180.0	C4—S1—Cd1^iv^	97.18 (11)
N5^i^—Cd1—N1	89.51 (9)		
			
C8—C2—C5—C6	0.6 (5)	N1—C1—N2—N3	0.1 (3)
C2—C5—C6—N4	−1.0 (5)	N1—C1—N2—C6	177.3 (3)
C2—C5—C6—N2	178.3 (3)	N4—C6—N2—C1	−1.0 (4)
C5—C2—C8—C7	0.0 (5)	C5—C6—N2—C1	179.6 (3)
N4—C7—C8—C2	−0.3 (5)	N4—C6—N2—N3	175.9 (2)
Cl1—C7—C8—C2	−179.4 (3)	C5—C6—N2—N3	−3.5 (4)
N2—C1—N1—C3	−0.1 (3)	N1—C3—N3—N2	−0.1 (4)
N2—C1—N1—Cd1	−176.61 (18)	C1—N2—N3—C3	0.0 (3)
N3—C3—N1—C1	0.1 (4)	C6—N2—N3—C3	−177.5 (3)
N3—C3—N1—Cd1	176.6 (2)	C5—C6—N4—C7	0.8 (4)
N5^i^—Cd1—N1—C1	−1.9 (3)	N2—C6—N4—C7	−178.6 (2)
N5—Cd1—N1—C1	178.1 (3)	C8—C7—N4—C6	−0.1 (4)
S1^ii^—Cd1—N1—C1	−93.2 (2)	Cl1—C7—N4—C6	179.1 (2)
S1^iii^—Cd1—N1—C1	86.8 (2)	N1—Cd1—N5—C4	−14.4 (5)
N5^i^—Cd1—N1—C3	−177.6 (3)	N1^i^—Cd1—N5—C4	165.6 (5)
N5—Cd1—N1—C3	2.4 (3)	S1^ii^—Cd1—N5—C4	−104.4 (4)
S1^ii^—Cd1—N1—C3	91.1 (3)	S1^iii^—Cd1—N5—C4	75.6 (4)
S1^iii^—Cd1—N1—C3	−88.9 (3)		

Symmetry codes: (i) −*x*, −*y*+2, −*z*+2; (ii) *x*−1, *y*, *z*; (iii) −*x*+1, −*y*+2, −*z*+2; (iv) *x*+1, *y*, *z*.
